# Dominant hemisphere functional networks compensate for structural connectivity loss to preserve phonological retrieval with aging

**DOI:** 10.1002/brb3.495

**Published:** 2016-07-27

**Authors:** Smriti Agarwal, Emmanuel A. Stamatakis, Sharon Geva, Elizabeth A. Warburton

**Affiliations:** ^1^ Stroke Research GroupAddenbrooke's HospitalUniversity of CambridgeR3, Box 83, Hills RoadCambridgeCB2 2QQUK; ^2^ Division of AnaesthesiaAddenbrooke's HospitalUniversity of CambridgeBox 93, Hills RoadCambridgeCB2 2QQUK; ^3^ Developmental Cognitive Neuroscience UnitUCL Institute of Child Health30 Guilford StreetLondonWC1N 1EHUK

**Keywords:** Aging, DTI, functional connectivity, language, phonology, PPI, word retrieval

## Abstract

**Introduction:**

Loss of hemispheric asymmetry during cognitive tasks has been previously demonstrated in the literature. In the context of language, increased right hemisphere activation is observed with aging. Whether this relates to compensation to preserve cognitive function or dedifferentiation implying loss of hemispheric specificity without functional consequence, remains unclear.

**Methods:**

With a multifaceted approach, integrating structural and functional imaging data during a word retrieval task, in a group of younger and older adults with equivalent cognitive performance, we aimed to establish whether interactions between hemispheres or reorganization of dominant hemisphere networks preserve function. We examined functional and structural connectivity on data from our previously published functional activation study. Functional connectivity was measured using psychophysiological interactions analysis from the left inferior frontal gyrus (LIFG) and the left insula (LINS), based on published literature, and the right inferior frontal gyrus (RIFG) based on our previous study.

**Results:**

Although RIFG showed increased activation, its connectivity decreased with age. Meanwhile, LIFG and LINS connected more bilaterally in the older adults. White matter integrity, measured by fractional anisotropy (FA) from diffusion tensor imaging, decreased significantly in the older group. Importantly, LINS functional connectivity to LIFG correlated inversely with FA.

**Conclusions:**

We demonstrate that left hemispheric language areas show higher functional connectivity in older adults with intact behavioral performance, and thus, may have a role in preserving function. The inverse correlation of functional and structural connectivity with age is in keeping with emerging literature and merits further investigation with tractography studies and in other cognitive domains.

## Introduction

Aging is accompanied by a complex pattern of structural and functional changes in the brain and has a differential effect on cognitive domains and between individuals (Christensen et al. 1999; Davis et al. 2011; Fling and Seidler 2012; Kerchner et al. 2012; Lebel et al. 2012). The HAROLD (Hemispheric Asymmetry Reduction in Older adults) model (Cabeza 2002) suggests that functional brain activity becomes less asymmetrical with age. Dedifferentiation of function and compensation, are two divergent mechanistic hypotheses for this model. The dedifferentiation hypothesis interprets the reduction in asymmetry as loss of ability to recruit specialized neural regions. Alternatively, the compensation hypothesis proposes that contralateral recruitment counteracts cognitive decline. Another prominent theory of aging and cognition is Salthouse's (2010) theory of “cognitive slowing” which states that there is a gradual decline of cognitive abilities with increasing age. However, this slowing may be variable between domains and at different steps of processing. Slowing of neural responses in the aging brain have been demonstrated previously and in the context of preserved cognitive ability, may be more widespread (Galdo‐Alvarez et al. 2009), which is in keeping with the basic conception of the HAROLD model. Wider brain activation with aging may also be interpreted as inefficiency or as recruitment of other, possibly nonlinguistic, cognitive abilities (Grady 2008). A recent comprehensive review of published studies regarding aging and language preservation (Shafto and Tyler 2014) suggests that core linguistic processes involve similar systems in the older and younger adults, raising the possibility that right hemisphere activation during left localized language faculties, such as phonology, may represent lack of functional specificity rather than a compensatory response.

Transcallosal white matter tracts connect homologous cortical regions and mediate information transfer and integration between hemispheres (Aboitiz 1992; Fling et al. 2011a; Fling and Seidler 2012). Loss of callosal fibers has been consistently demonstrated with increasing age (Davis et al. 2011; Fling and Seidler 2012; Kerchner et al. 2012; Lebel et al. 2012). Thus, preservation of certain cognitive abilities is likely to be mediated by alterations in functional interactions between and within hemispheric regions relevant to the cognitive domain of interest. This has led to the “less wiring, more firing” hypothesis, implying that higher functional network activation may mediate preserved function in older adults despite loss of white matter integrity as shown recently in the domains of executive function and memory (Daselaar et al. 2015). Understanding of language pathways in the human brain has significantly evolved from the classic Wernicke–Lichtheim–Geschwind model to incorporate knowledge of cortical areas other than the traditional perisylvian language areas [e.g., the role of nonlinguistic cognitive processes, especially the contribution of the attentional network, is being increasingly recognized in language models (Hagoort 2013)] and white matter pathways connecting cortical areas (Poeppel and Hickok 2004; Friederici et al. 2011; Rijntjes et al. 2012; Friederici and Gierhan 2013). Thus, changes in white matter integrity with aging are important to characterize in order to fully understand functional network changes in older adults that underlie preserved linguistic function.

In this study, we reanalyzed data from our previously published study of healthy younger and older adults, which explored functional activations during a word rhyming and picture recognition task (Geva et al. 2012). The aim of this study was to focus on word retrieval per se and examine functional connectivity to describe the role of hemispheric networks underlying preserved retrieval. Unlike the previous study, this work studied functional connectivity rather than functional activations and importantly, examined the relationship of functional connectivity to white matter structure, a key aspect that was not explored by the previous work.

Age‐related right hemisphere activation during language tasks does not consistently support the compensation or dedifferentiation hypotheses in the HAROLD model. For instance, Wierenga et al. (2008) showed that right Inferior Frontal Gyrus (RIFG) activation, with concurrent decrease in right Precentral Gyrus activation, was associated with preserved picture naming. Similarly, Tyler et al. (2010a) found right frontotemporal activation together with preserved syntactic processing. Contrastingly, Meinzer et al. (2009) found RIFG and right Middle Frontal Gyrus activations together with poorer performance on semantic fluency, but no effect on phonological fluency. Thus, functional brain activations alone do not entirely explain age‐related neural reorganization. Interactions between brain regions are likely to fundamentally underlie age‐related neuroplasticity.

Functional connectivity analysis allows the examination of synchronization between brain regions (Horwitz 2003; Fox and Raichle 2007) and can help disentangle the neural underpinnings of successful cognitive aging. We employed one such approach, namely, psychophysiological interactions (PPI) analysis (Cabeza et al. 1997; Friston et al. 1997) to explore network interactions of right and left hemispheric areas during a rhyme judgment task. We have previously applied this task in our studies of inner speech (Geva et al. 2011a,b) and related publication on aging (Geva et al. 2012).

Our previous study (Geva et al. 2012), found an age‐related increase in RIFG activation during a word rhyming task which may have a role in inhibiting errors. In that study, we suggested that RIFG is not related to core linguistic processes during the rhyming task such as grapheme‐to‐phoneme conversion, for example, but rather to an executive ability to monitor behavior. This is what partly motivated this study, as we aimed to determine younger/older adult network connectivity differences rather than activity in individual regions. Also there is suggestion in the broader literature that right hemisphere may not host phonological representations, but may have a role in context processing. We also focused, in detail, on functional connectivity and its relationship with white matter structure.

Using two different contrasts, that is, words > rest and words > baseline task, we aimed to disentangle age effects on overall task performance versus phonological processing per se, at the network level.

We examined the following hypotheses:

1. Our previous related study revealed increased RIFG activation during the word rhyming task (Geva et al. 2012). If this activation was compensatory, we would expect to find higher overall functional connectivity of this region in older participants compared to younger participants. In addition, RIFG functional connectivity would be expected to positively correlate with behavioral performance.
2. Based on previously published literature on phonology, left hemispheric regions, particularly the left insula (LINS), have been implicated in phonological processing and aging (Wise et al. 1999; Cereda et al. 2002; Shafto et al. 2007). Given the evidence for age‐related white matter changes (Nusbaum et al. 2001; Stamatakis et al. 2011), if these left hemispheric regions indeed preserve function with increasing age, we expect their functional connectivity to be higher in older adults. Also, functional connectivity would be expected to positively correlate with behavioral performance.
3. Since white matter (WM) integrity changes with age (Wierenga et al. 2008; Stamatakis et al. 2011), we expected to find lower white matter fractional anisotropy (FA) as measured using Diffusion Tensor Imaging (DTI), especially in transcallosal tracts. We also expected a negative relationship between functional connectivity measures and white matter integrity, if indeed the network level changes outlined compensate to maintain function.

## Materials and Methods

### Subjects

The dataset is common to our previous study discussed in previous sections. Two groups participated in the study: 12 young adults (four men and eight women; age range = 21–34 years, mean age = 24.6 ± 4.5 years; mean number of years of education = 18.1 ± 2.1) and 19 older adults (eight men and 11 women; age range = 55–71 years, mean age = 64.1 ± 4.8 years; mean number of years of education = 15.1 ± 2.9). All participants had no history of neurological, psychiatric, or language disorders, as verified using a medical questionnaire, were right‐handed as measured by the Edinburgh handedness inventory (Oldfield 1971) and native speakers of British English. All participants completed a task measuring nonverbal IQ on Raven's Progressive Matrices (Basso et al. 1987). Although the younger participants had significantly more years of education (independent samples *t* test, *t* = 3.10, *P* = 0.004), the two groups did not differ in their performance on the Raven's Progressive Matrices (independent samples *t* test, *t* = 0.74, *P* = 0.47). This study was approved by the Cambridge Research Ethics Committee; all participants gave written, informed consent.

### Imaging data acquisition

#### fMRI

Imaging was performed using a 3T Siemens (Erlangen, Germany) Magnetom Trio MRI scanner at the Wolfson Brain Imaging Centre in Cambridge. Two hundred and thirty four whole‐brain T2*‐weighted EPIs (slice thickness = 3.75 mm, inplane resolution 3 × 3 mm, 32 axial slices, sequence: interleaved ascending, repetition time = 2 sec, echo time = 30 ms, flip angle = 78°; matrix = 64 × 64, field of view = 192 × 192 mm) were acquired. The first six volumes were treated as dummy pulses and were discarded to allow for signal equilibrium effects. A magnetization‐prepared rapid acquisition gradient echo (MPRAGE) scan was also acquired (repetition time = 2.3 sec, echo time = 2.98 sec, inversion time =900 ms, flip angle = 9°, field of view = 240 × 256 mm, sagittal plane; slice thickness = 1 mm; 176 slices).

#### DTI

The diffusion‐weighted imaging (DWI) acquisition sequence was a single‐shot spin echo planar imaging (EPI) sequence, with echo time (TE) = 98 ms and repetition time (TR) = 8300 ms. Sixty‐three contiguous 2‐mm‐thick axial slices were obtained, covering the whole brain. Diffusion sensitizing gradients were applied in 12 gradient directions repeated for each of 5 b‐values equally spaced between 0 and 1500 mm^2^/sec, using a double‐spin echo sequence to reduce eddy current effects. The use of multiple b values for equivalent number of gradient directions has been shown to improve contrast to noise ratio in diffusion‐weighted white matter imaging (Correia et al. 2011). The field of view was 19.2 cm, and the acquisition matrix size was 96 × 96, giving a voxel size of 2 × 2 × 2 mm. Acquisition time was 9 min 26 sec.

#### fMRI task

To create stimuli for the rhyme judgment task, 130 nouns were chosen, which create pairs of rhymes according to the Oxford British Rhyming Dictionary (Upton and Upton 2004) as described by our group previously (Geva et al. 2011b, 2012). The words' ending in each pair differed in their orthography, so that participants could not make the rhyme judgment based on orthography alone. The final list contained 36 word pairs, out of which 26 pairs rhymed (about 70%) and the rest 13 did not rhyme (about 30%). These words were presented as written words in a block. A potential methodological problem was that if each word appeared once in a rhyming pair and once in a nonrhyming pair, participants could develop a strategy whereby they might not use language access, but rather remember that if they saw the word before in a rhyming pair, it is necessarily a nonrhyming pair this time, and vice versa. To avoid this, some words appeared only in rhyming pairs (once or twice) and some words appeared once in a rhyming pair and once in a nonrhyming pair. This way participants could not predict the correct answer from the previous appearance of the word.

A high level baseline condition employing a visual similarity task, was chosen to control for activation associated with: (1) Visuo–spatial processing, (2) Comparison between two items, (3) Decision making, and (4) Motor response. Meaningless symbols were used and participants had to indicate whether two images were identical or not. The stimuli did not resemble letters, therefore reducing the risk of inducing spontaneous naming. For the words baseline, 26 symbol strings were used. In 26 pairs the two symbols were identical, and in additional 10 pairs the two symbols were different. In the latter pairs, a string of symbols was paired with stars (*** or ****) to create nonidentical pairs. The side on which the stars and the symbols appeared was counterbalanced across trials.

The majority of words were relatively high frequency as the same words were used for a picture matching task in our previous related publication which requires them to be high frequency, together with high imageability.

Word retrieval can occur via the grapheme‐to‐phoneme conversion or lexical route. We called the process “phonological word retrieval” to emphasize that the task cannot be performed without correct retrieval of the phonological form of the word. If a participant attempted to solve the task based on orthography alone, without accessing phonology, the task would not be performed correctly, as some pairs contained words with irregular spelling. The task was designed this way exactly in order to prevent an exclusive orthography‐based strategy.

Participants performed a rhyme judgment task as reported in our previous study (Geva et al. 2012), on 36 written word pairs, out of which 26 pairs rhymed (about 70%) and 10 did not (about 30%) with mean number of letters = 4.25 ± 0.8, range = 3–6; mean number of phonemes = 3.10 ± 0.8, range = 1–4; mean number of syllables = 1.03 ± 0.2, range = 1–2. The baseline condition was a visual similarity task containing strings of meaningless symbols (e.g.,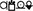) where participants had to indicate whether two visual stimuli were identical using a button press. Participants first practiced the task outside the scanner. The practice blocks were shorter, containing 20 pairs, 10 of which were rhyming pairs and 10 were nonrhyming pairs. To reduce subvocal articulation and subsequent scanning artifacts, participants practiced the task until they managed to avoid vocalization and articulatory movements. In each trial of the rhyme judgment, participants were presented with two words for 7.3 sec and had to indicate, within this time frame, whether the words rhymed, by pressing one of two buttons. In the baseline condition, subjects were asked to indicate whether two strings of symbols were identical. In each trial, the words “yes” and “no,” together with a ✓ and an Χ, respectively, appeared at the bottom of the screen, to remind participants that the left button corresponds to a “yes” answer and the right button corresponds to a “no” answer. After the trial, a fixation cross appeared for 1.5 sec, and after every third trial a longer fixation cross appeared for 13 sec. In the last 2 sec of the long fixation period, the color of the cross turned to red, alerting the participant that the next trial was starting. Each run lasted about 7 min. Participants were able to rest between runs. The stimuli were presented using E‐Prime^®^ (version 1.2; Psychology Tools, Inc., Pittsburgh, PA), in blocks of similar stimulus, to maximize design efficiency. The order of blocks was randomized and counterbalanced between participants. The order of trials was pseudorandomized, making sure that the same word did not repeat in two consecutive trials. Stimuli were projected onto a black screen with a resolution of 1024 × 768 pixels.

#### fMRI processing

fMRI data were preprocessed using SPM8 (Wellcome Trust Centre for Neuroimaging, London, UK, www.fil.ion.ucl.ac.uk/spm) implemented in the Matlab (Mathworks^®^, Natick, MA) environment (2006b). Motion correction was performed using the realign function, by first registering all fMRI images to the first image (after excluding the six dummy scans), and then registering all images to the mean. Coregistered (to the mean fMRI) structural images were segmented into GM, WM, and CSF probability maps, and spatially normalized to Montreal Neurological Institute (MNI) space, using the unified segmentation–normalization algorithm. This procedure combines tissue segmentation, bias correction, and spatial normalization in a single unified model (Ashburner and Friston 2005). Normalized GM images were visually inspected for quality of the segmentation–normalization process. The spatial normalization parameters were then applied to the functional images. The voxel size in the normalized functional images was resampled to 2 × 2 × 2 mm. Functional images were spatially smoothed using an 8 mm *full width at half maximum* (FWHM) Gaussian kernel. At the first level statistical modeling, the different conditions, as well as correct and incorrect responses in each task, were modeled as separate conditions/regressors in a general linear model. Six affine motion parameters from the realignment stage of preprocessing were added as regressors of no interest in the model.

**Figure 1:**
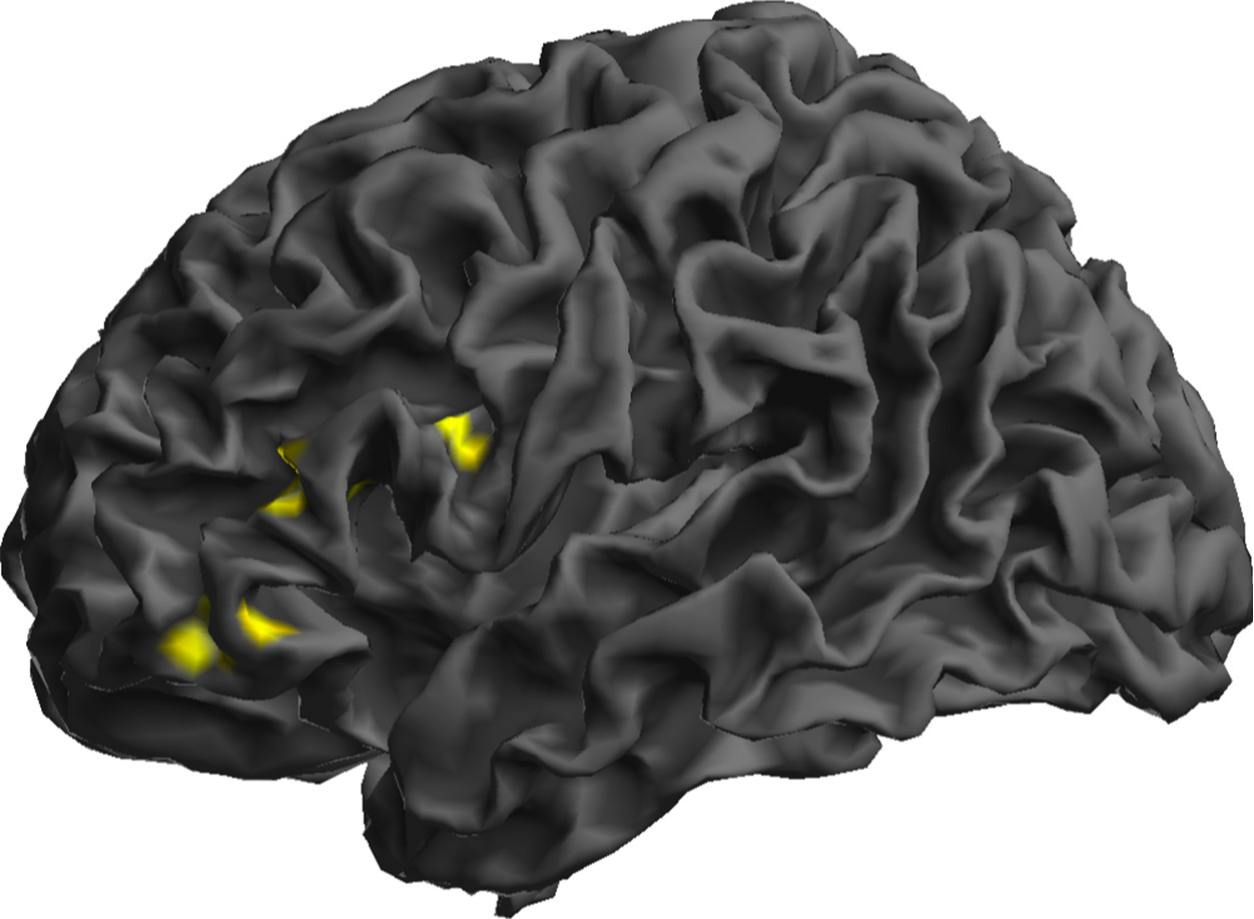
Second level subtractive analysis for words > baseline contrast for old > young groups (*P* < 0.005, uncorrected).

Second level analysis for the words > rest has been reported previously (Geva et al. 2012). Additionally, we performed an exploratory second level analysis for words > baseline to explore the role of left hemisphere language areas based on published literature on phonology. We used a lower statistical threshold (*P* < 0.005) to identify seed regions for PPI analysis, given the preexisting evidence regarding role of these areas, particularly the left insula (Wise et al. 1999; Cereda et al. 2002; Shafto et al. 2007; Papoutsi et al. 2011), in phonological processing. The exploratory analysis was performed with the single aim of picking seed regions for connectivity analysis and is not otherwise applied for our hypothesis testing. These results at an exploratory threshold of *P* < 0.005 uncorrected are shown in Figure 1.

#### PPI

A psychophysiological interaction analysis investigates functional integration where the interaction of one brain region to another is modulated by the experimental psychological task (Friston et al. 1997). Voxel time series were extracted from each seed region of interest (ROI as a sphere with a radius of 4 mm) for each participant and summarized as the first principal component across all voxels within the seed ROI sphere. Vectors representing the psychological factor (task) were convolved with the extracted time series to create the PPI regressors, for each of the seed regions. In keeping with the contrast used for subtractive analysis for fMRI activations, these were words > rest for RIFG following on from our previous related publication (Geva et al. 2012) and words > baseline condition for LIFG and LINS. The PPI regressors together with the seed ROI time series, the experimental task vector and the six movement parameters as effects of no interest, were then entered into a general linear model (PPI‐GLM) for every participant.

For each seed ROI, we created a contrast image for the main effect of PPI for each group and then used an independent samples *t*‐test to test for differences between the older and younger age groups. While the connectivity changes in the younger group may not necessarily have been performance related, our aim was to assess whether any differences in functional connectivity between the older and younger groups contribute to preservation of performance, as well as, examining the role of hemispheric networks involved in phonological retrieval in either group.

All fMRI results are reported at a cluster corrected threshold of *P* < 0.05 with clusters of at least 20 voxels, unless otherwise specified.

### Behavioral data and correlations with PPI

For each subject, reaction time (RT) and error rate (%) were noted. These were available for the task as well as the baseline conditions. Mean values with standard deviations are reported and compared between the age groups. At the group level, a variable indicating the effect size of the interaction from the PPI analysis was extracted for each cortical area found to interact with the seed ROI. This was entered into a correlation analysis with RT. The error rates were very low for both groups, hence RT was used as the main behavioral measure for examining relationship with PPI effect size. In an ancillary analysis, error rate was used as shown in supplementary tables.

Statistical analysis for behavioral data was performed using IBM SPSS^®^ (version 19 for Macintosh; NY, USA) and Microsoft Excel^®^.

In order to provide a map of which area's functional connectivity predicts behavioral performance, we applied a parametric RT model to the PPI analysis from all three seed regions.

Given the large number of variables for correlations, a stricter statistical threshold of *P* < 0.001 (2‐sided) was used for determining if behavioral performance correlated with PPI effect sizes.

### DTI processing

In aging studies, fractional anisotropy (FA) changes display high sensitivity to changes in cognitive function (Nusbaum et al. 2001; Schiavone et al. 2009). Here, we measured FA as a marker of underlying structural white matter changes that may be related to altered connectivity in brain activations between regions relevant to language processing.

Our DTI acquisition protocol allowed for images to be acquired within a relatively short scanning period, which is optimal for obtaining radial diffusivity measures and FA (Correia et al. 2011). However, given that the number of directions of gradients applied was only 12, the data acquired cannot be meaningfully used for tractography (Correia et al. 2009).

FA images were derived from DTI data using the FSL^®^(fMRIB, Oxford, UK) FDT processing pipeline. All images were eddy current corrected, and a diffusion tensor model was fitted to each voxel using a binary brain mask extracted using the Brain Extraction Tool (BET) from FSL FDT.

The B0 images were spatially normalized to the EPI template in SPM8^®^ (Wellcome Functional Imaging Laboratory, London, UK) and normalization parameters were applied to the raw FA images. Furthermore, a study and modality‐specific FA template was created (Stamatakis et al. 2011) to which all raw FA images were normalized prior to group analysis. Each participant's FA images were visually assessed to ensure successful spatial normalization to this template before group analysis. A voxelwise comparison was subsequently performed using SPM8, with a two sample *t‐test*, between the older and younger age groups. We masked the results using a white matter mask, derived by binarizing (thresholding at 0.2) the SPM a priori white matter probabilistic image. We report results for clusters that survived corrections for multiple comparisons at *P* < 0.05 and had >20 voxels unless otherwise specified in the text.

Unthresholded figures are available from Neurovault at link http://neurovault.org/collections/1244/.

## Results

### Behavioral results

Average RTs for the word task were 1594 ± 287 ms and 1567 ± 437 ms for the older and young adults, respectively. For the baseline task, average RTs were 1347 ± 425 and 1115 ± 374 for the older and young adults, respectively. On the word task, the older participants had an average error rate of 0.44 ± 1.0%, whereas the younger participants had an average error rate of 1.2 ± 1.9%. For the baseline task, the older participants had an average error rate of 0.15 ± 0.64%, whereas the younger participants had an average error rate of 0.76 ± 1.3%. The word task had longer RT than baseline condition for both groups (independent samples *t*‐test, *P* value 0.04 for the older group and 0.01 for the younger group). The error rate was not different for either group between the task and baseline condition (independent samples *t*‐test *P* value, 0.9 for the older group and 0.4 for the younger group. The two groups did not significantly differ in RT or error rate (independent samples *t*‐test >0.1) for the task as well as baseline condition.

### fMRI activation results

As described previously (Geva et al. 2012), RIFG pars orbitalis (POrb) activation was higher for older than younger participants for the words > rest contrast (x = 40,y = 36,z = −2, Z score = 5.10; *P* < 0.05 FWE corrected).

In an exploratory analysis performed with the intention to define left hemisphere language‐related seed regions for the words>baseline contrast (uncorrected voxel threshold of *P* < 0.005), comparing activations between groups (old > young), older participants had greater activation in the LIFG pars triangularis (PTri, x = −46, y = 26, z = 8, Z score = 4.60) and left insula (LINS x = −30, y = 20, z = −14, Z score = 3.47). These results are shown in Figure 1. This is in keeping with observations from other studies that preserved left hemispheric function (Wierenga et al. 2008), particularly in the left insula (Shafto et al. 2010), may have a role in preserving performance on phonological tasks in older individuals.

### PPI results for the three seed regions of interest

With the aim of studying age‐related functional connectivity effects, seed regions showing greater activations in the older group were selected as above. We use the words > rest contrast to uncover overall task performance effects and the words > baseline contrast for effects specific to phonological processing. For the words > rest contrast, we used the RIFG as seed region (x = 40,y = 36,z = −2) and for the words > baseline contrast, we used the LIFG (x = −46, y = 26, z = 8) and the LINS (x = −30, y = 20, z = −14) following on from the activation analysis.

RIFG (x = 40,y = 36,z = −2) connectivity results during the words > rest contrast are shown in Table 1. For the older group, RIFG significantly connected to left middle frontal gyrus, right postcentral gyrus and RIFG POrb; whereas in the younger group, connectivity was significant for RIFG PTri, LIFG PTri, and left middle frontal gyrus. In a group comparison aimed to uncover age effects, the younger group had higher connectivity to bilateral inferior frontal areas on both sides. Also, there was higher connectivity for the younger group between the RIFG and the left rolandic operculum. No negative interactions were found, even at *P* < 0.001 voxel level uncorrected threshold, for the RIFG seed region for either of the two groups. Thus, RIFG connected to bilateral inferior frontal regions in both groups, but connectivity was higher in younger adults.

**Table 1:** PPI from RIFG at a cluster threshold, *P* < 0.05 (FWE corrected) and clusters with ≥20 voxels

	Seed region	Brain region	x	y	z	Peak t value	Peak z score	RT versus PPI r (p)	Cluster size
All	40, 36, −2	RIFG POrb	40	34	−2	12.67	7.25		162,304 (contiguous)
		Left middle frontal	−40	42	20	12.20	7.19		162,304 (contiguous)
		LIFG P Tri	−42	34	16	12.20	7.19		162,304 (contiguous)
Old		Left middle frontal	−40	42	20	9.57	6.33	−0.12 (0.631)	129,286 (contiguous)
		Right postcentral	20	−38	52	9.17	6.18	0.08 (0.744)	188 (contiguous)
		RIFG POrb	40	36	−2	9.00	6.12	−0.16 (0.505)	188 (contiguous)
Y		RIFG PTri	40	34	0	10.16	6.53	0.33 (0.317)	64
		LIFG PTri	−42	34	16	9.99	6.24	−0.06 (0.872)	85,992 (contiguous)
		Left middle frontal	−34	36	18	9.27	6.22	0.03 (0.939)	85,992 (contiguous)
Y > O		RIFG PTri	40	32	0	4.83	4.09	−0.03 (0.882)	788 (contiguous)
		RIFG PTri	52	40	6	3.89	3.45		788 (contiguous)
		RIFG POp	60	22	20	3.89	3.45	−0.19 (0.319)	788 (contiguous)
		LIFG PTri	−36	34	18	4.74	4.03	−0.10 (0.607)	936 (contiguous)
		LIFG POp	−56	10	26	3.87	3.44	−0.19 (0.320)	936 (contiguous)
		Left rolandic operculum	−58	6	8	3.73	3.33	−0.17 (0.371)	936 (contiguous)

Connectivity from LIFG (x = −46, y = 26, z = 8) seed region is shown in Table 2. For the older group, LIFG connected to RIFG, left middle frontal gyrus, LIFG POrb, right inferior temporal area, right cerebellum, right caudate, right precentral gyrus, and left thalamus. For the younger group, no significant connectivity was found at the prespecified FWE corrected threshold; at an uncorrected voxel threshold (*P* < 0.001), connectivity between LIFG and other left‐sided regions was noted in the younger group. No negative interactions nor any significant group differences were found even at *P* < 0.001 uncorrected threshold.

**Table 2:** PPI from LIFG at a cluster threshold 0.05 (FWE corrected) and clusters with ≥20 voxels

	Seed region	Brain region	x	y	z	Peak t value	Peak z score	RT versus PPI r (p)	Cluster size
All	−46,26,8	RIFG POrb	46	48	−4	4.77	4.07	0.07 (0.709)	451
		Cluster to RIFG (P opercularis)	24	−10	34	4.15	3.65	0.01 (0.981	5510
		Cluster to LIFG (P opercularis)	−28	6	28	4.08	3.60	−0.20 (0.294)	595
		Left middle frontal	−24	40	−16	4.35	3.79	0.03 (0.885)	671 (contiguous)
		Cluster to L middle frontal	−26	42	0	3.81	3.40		
		LIFG PTri	−36	40	4	3.78	3.98	0.12 (0.517)	671 (contiguous)
Old		RIFG POrb	46	44	−4	5.47	4.14	0.19 (0.446)	1980 (contiguous)
		RIFG PTri	44	36	28	5.18	4.00	0.23 (0.336)	1980 (contiguous)
		RIFG PTri	42	28	24	4.99	3.91		
		Lt middle frontal (orb)	−40	52	−6	5.23	4.03	−0.06 (0.801)	691 (contiguous)
		LIFG POrb	−48	44	−2	4.46	3.61	0.05 (0.825)	691 (contiguous)
		Rt cerebellum	16	−86	−28	4.65	3.72	0.05 (0.841)	318 (contiguous)
		Rt cerebellum	16	−70	−26	3.89	3.27		
		Rt inferior temporal	46	−54	−22	4.99	3.57	−0.18 (0.466)	434
		Lt thalamus	−2	−12	6	4.59	3.69	0.14 (0.582)	1062 (contiguous)
		Rt precentral	50	4	48	3.92	3.29	0.12 (0.630)	1062 (contiguous)
		Rt Caudate	18	−8	22	3.81	3.22	0.10 (0.687)	1062 (contiguous)
Y		None at above threshold							
	At *P* < 0.001	Left insula	−32	10	16	4.43	3.22	0.05 (0.877)	38
	At *P* < 0.001	Cluster to LIFG P opercularis	−24	12	34	4.29	3.13	0.25 (0.466)	66
	At *P* < 0.001	Left middle frontal orbitalis	−28	42	−20	4.01	3.02	0.15 (0.662)	48
	At *P* < 0.001	Left sup medial frontal	−10	58	30	4.00	3.02	−0.10 (0.779)	
Y > O		None at above or *P* < 0.001							
O > Y		None at above or *P* < 0.001							

**Figure 2:**
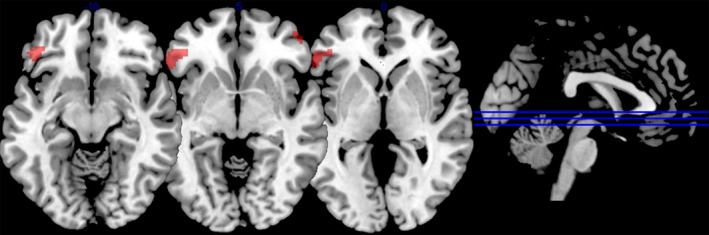
Left insula PPI for older > younger adults at *P* < 0.001 uncorrected voxel threshold with clusters >20 voxels.

From the LINS (x = −30, y = 20, z = −14) seed region, connectivity results are shown in Table 3. For the older group, left insula connected to both left and right hemispheric areas, namely, LIFG POrb and LIFG PTri, left and right middle frontal gyri, right superior frontal gyrus and right caudate region. No significant connectivity was seen in younger adults from left insula. In a subtractive analysis between the two groups using a two sample *t*‐test, no differences were found at the FWE corrected threshold, but at *P* < 0.001 uncorrected threshold, higher insular connectivity for the older group was found with LIFG POrb, right middle frontal gyrus, LINS, left middle frontal, and left fusiform gyrus (Fig. 2). No significant negative interactions were found.

**Table 3:** PPI from LINS −30,20,−14 at a cluster threshold 0.05 (FWE corrected) and clusters with ≥20 voxels

	Seed region	Brain region	x	y	z	Peak t value	Peak z score	RT versus PPI r (p)	Cluster size
All	−30,20,−14	None at above threshold							
Old		LIFG PTri	−42	38	2	5.78	4.29	−0.33 (0.172)	712 (contiguous)
		Left middle frontal	−38	58	6	5.76	4.28	−0.22 (0.375)	712 (contiguous)
		LIFG POrb	−40	38	−10	4.79	3.80	−0.36 (0.132)	712 (contiguous)
		Rt middle frontal orb	38	58	−2	5.30	4.06	0.00 (0.999)	452
		Rt sup frontal orb	20	20	−12	4.49	3.69	−0.10 (0.693)	222 (contiguous)
		Rt caudate	12	26	−6	3.85	3.25	0.04 (0.878)	222 (contiguous)
Y		None at above or *P* < 0.001							
O > Y		None at above threshold							
	At *P* < 0.001	LIFG POrb	−44	36	−10	5.02	4.20	−0.20 (0.297)	517 (contiguous)
	At *P* < 0.001	Left insula	−34	22	−8	3.56	3.21	−0.22 (0.244)	517 (contiguous)
	At *P* < 0.001	Right middle frontal orbitalis	48	48	−6	4.89	4.12	−0.04 (0.818)	83
	At *P* < 0.001	Left fusiform	−36	−60	−16	3.33	3.03	−0.26 (0.164)	50 (contiguous)
	At *P* < 0.001	Left middle frontal	−48	42	20	3.26	2.97	0.12 (0.517)	50 (contiguous)

### Functional connectivity and behavioral performance

Parameter estimates for each region showing significant connectivity to the seed ROI were extracted; correlations between these parameter estimates and behavioral measures (RTs) are shown in Tables 1, 2, 3. No significant correlations were found. Correlations with error rates are shown in supplementary tables. Given the very low error rate, we did not apply this in our primary analysis.

**Figure 3:**
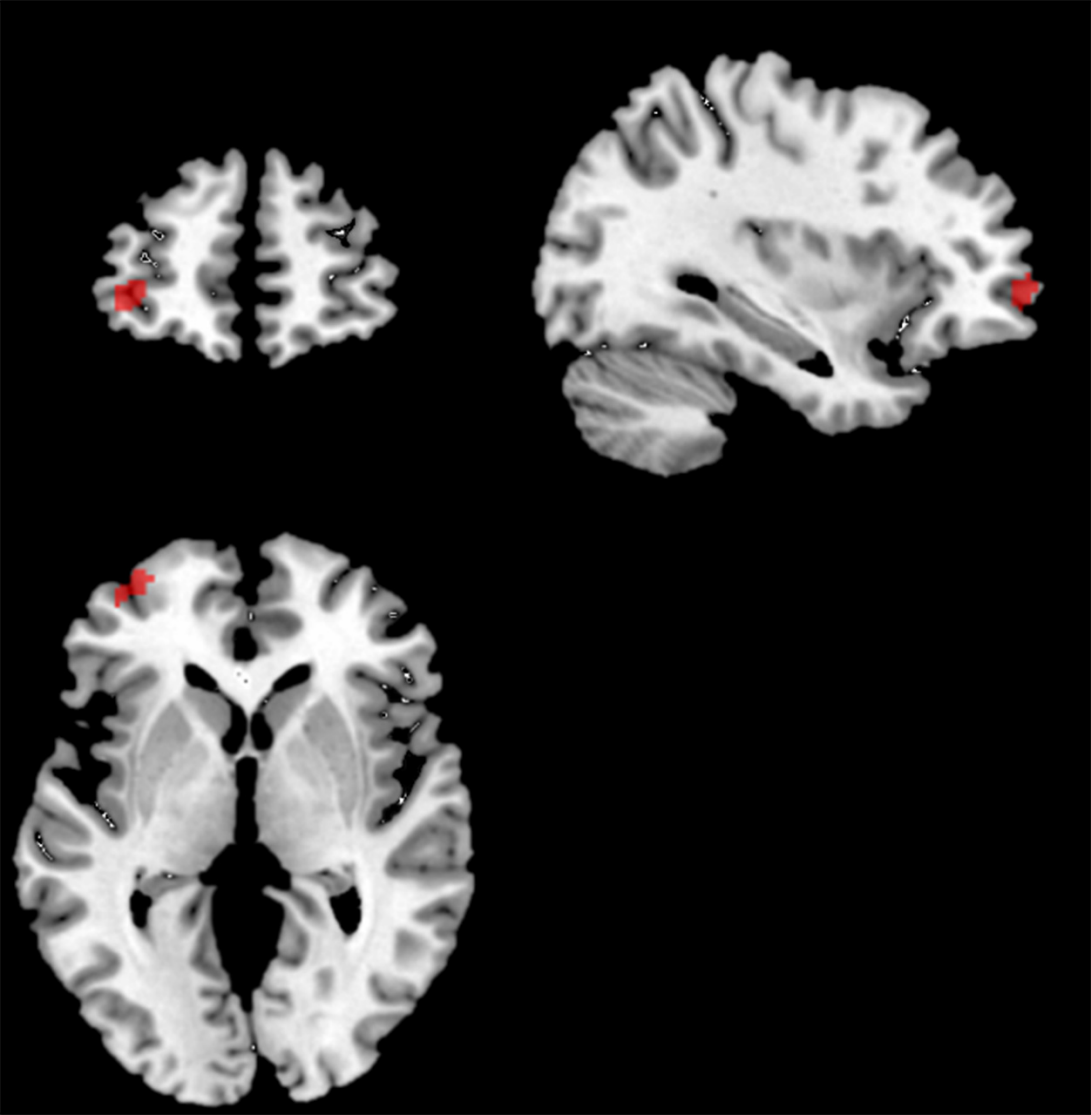
Parametric model with Reaction time (RT) applied to PPI analysis from left insula (−30, 20, −14) showing negative correlations (at *P* < 0.001 uncorrected and >20 voxels) between functional connectivity and RT.

We also applied a parametric model for RT to the three seed region PPI analyses. While no correlations were found at the FWE corrected threshold of *P* < 0.05 and over 20 voxels, we found a negative correlation in the LINS PPI model with RT as a regressor. The region of significance here was in the left frontal area (left middle frontal gyrus −34, 58, 2) as shown in Figure 3.

### DTI results

**Figure 4:**
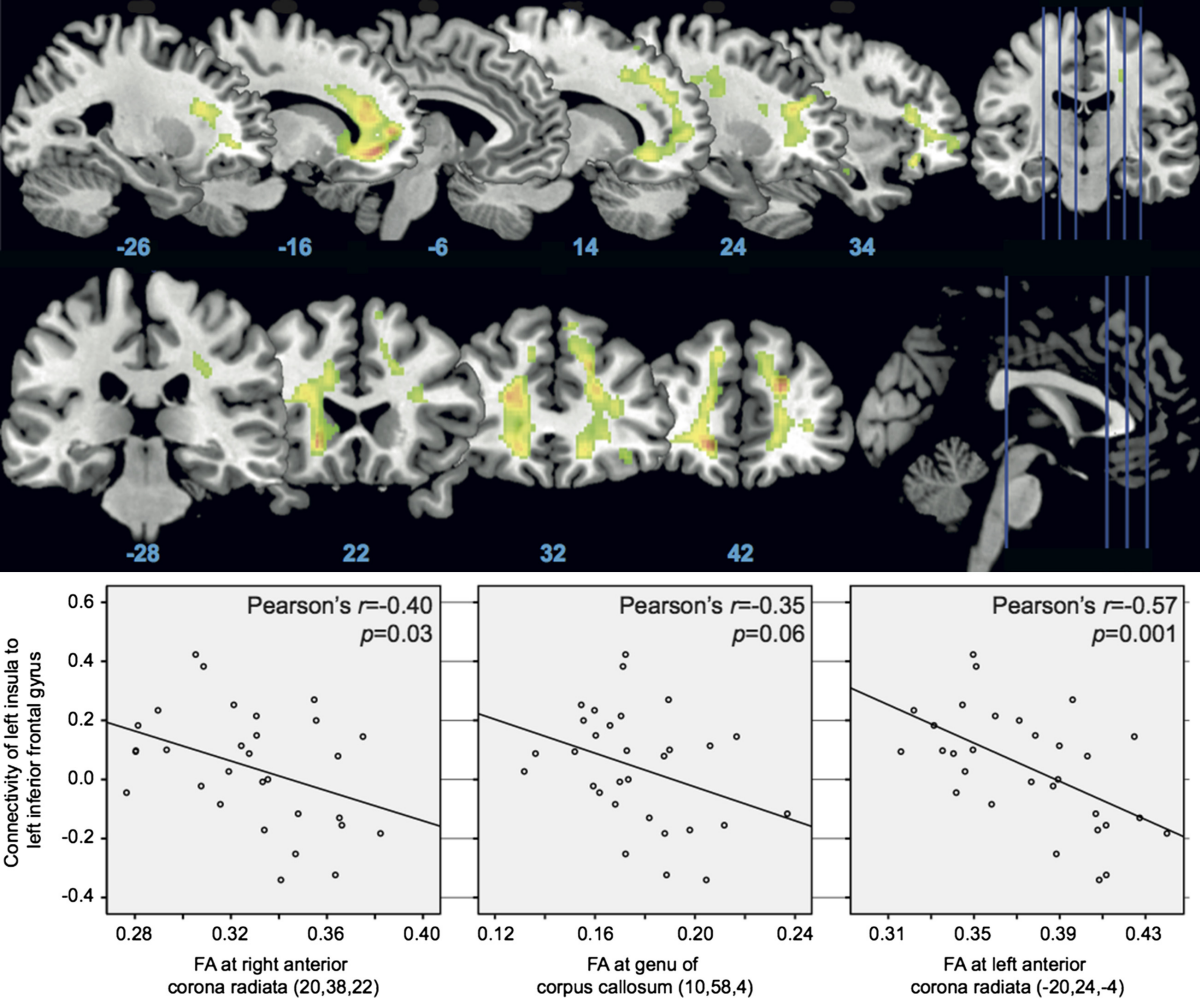
Fractional anisotropy (FA) measured on DTI for younger > older adults at *P* < 0.05 FWE corrected cluster threshold and clusters with >20 voxels (top panel). Correlation between left insula and left inferior frontal gyrus connectivity and FA (bottom panel).

Older participants had lower FA in bilateral anterior corona radiata and genu of the corpus callosum (Table 4, Fig. 4). This finding is in keeping with the postero‐anterior white matter atrophy gradient in aging (Nusbaum et al. 2001; Goh 2011). Given that LINS connectivity to LIFG was significantly related to lower error rates, we examined the relationship between this connectivity measure and mean FA extracted from the regions where the older adults had loss of white matter as shown in Table 4. There was a significant negative relationship between LINS to LIFG POrb functional connectivity and FA suggesting that increased functional connectivity may be compensating for age‐related alterations of white matter (Fig. 4). DTI measures did not correlate with task performance (*P* > 0.2 for Pearson's correlation coefficient for all six regions in Table 4).

**Table 4:** DTI analysis for young > old group comparisons at a cluster threshold *P* < 0.05 (FWE corrected) with clusters with 20 or more voxels; last column shows correlation of PPI between LINS and LIFG POrb with DTI

X	Y	Z	Anatomical region	T score	Z score	PPI versus DTI r (p)	Cluster size
20	38	22	Rt Anterior corona radiata	7.47	5.49	−0.40 (0.030)	1952
16	36	34	Rt Anterior corona radiata	6.95	5.25	−0.57 (0.001)	1952
10	58	4	Genu of corpus callosum	5.70	4.60	−0.35 (0.062)	1952
−20	24	−4	Lt anterior corona radiata	7.07	5.34	−0.57 (0.001)	2117
−16	42	−6	Lt anterior corona radiata	6.97	5.27	−0.55 (0.002)	2117
−16	50	6	Lt anterior corona radiata	6.39	4.98	−0.60 (0.0004)	2117

## Discussion

In this study, we applied PPI functional connectivity analysis to clarify hemispheric contributions to phonological retrieval that underlie preserved function with aging. We further explored the relationship between white matter changes using DTI and changes in network configuration measured by PPI. Previously, PPI analysis has been applied to both PET and fMRI data with examples including studies of aging and motor function (Rowe et al. 2006), motor networks (Kasahara et al. 2010), and memory networks in traumatic brain injury patients (Kasahara et al. 2011), syntactic performance in left hemisphere‐damaged patients (Papoutsi et al. 2011), and predictors of abnormal eating behaviors (Passamonti et al. 2009).

PPI analysis was applied to three seed regions of interest based on preexisting literature and our findings. The choice of connectivity seed regions in our study was motivated by two different contrasts, words > rest for RIFG and words > baseline for LIFG and LINS with an aim to uncover overall task performance effects versus task performance specific to phonological processing. In our previous work, we found higher activation RIFG during a word rhyming task (contrast words > rest); however, the role of this region in preserving phonology with age was not entirely clear. There was a suggestion from the data that this may have a role in error inhibition rather than core phonological processes (Geva et al. 2012). We, thus, applied this as one of our three seed regions to examine if connectivity of this region was greater in older adults in our cohort. LIFG and LINS have been previously implicated in phonological processing (Wise et al. 1999; Cereda et al. 2002; Shafto et al. 2007). To identify seed regions for these left hemispheric regions, we did an exploratory analysis for words > baseline contrast which has not been reported in our previous related study. At a lower statistical threshold (*P* uncorr <0.005), we were able to identify two seed regions in these areas (Fig. 1). The choice of these left hemispheric seeds was a‐priori, based on the theoretical motivation outlined in our hypotheses.

Below we discuss our three key hypotheses in order.

In relation to our first hypothesis, given the previous finding of greater RIFG activation in older adults during the rhyming task (Geva et al. 2012), we expected to find higher overall connectivity of this region in the older age group when compared with the younger group. This hypothesis was not confirmed. Although the RIFG connected to both right and left frontal areas in the older age group, overall connectivity was higher in the younger group. This is in keeping with previous literature on phonology, which supports the involvement of left rather than right hemispheric regions (Cereda et al. 2002; Shafto et al. 2007). There is also suggestion in previous literature (Vigneau et al. 2011) that the right hemisphere does not appear to support phonological ability and the activity noted may relate to nonlinguistic cognitive processes such as working memory and attention.

The finding that younger adults have higher RIFG functional connectivity for words > rest supports the idea that right hemispheric regions may be responsible for nonlinguistic components and thus, relate more to task performance overall. Moreover, since connectivity was lower in older adults, it further strengthens the idea that functional activation in this region supports functions other than phonology per se, at least in the older group. The dedifferentiation hypothesis in the HAROLD model interprets loss of hemispheric asymmetry with age as a decline in functional specialization rather than a compensatory mechanism. Our results for RIFG connectivity during phonological processing are, thus, more in keeping with the dedifferentiation, rather than compensation. We have previously shown that increased RIFG activation is related to preserved performance in participants more prone to making errors and may play a role in counteracting competing inputs (Stamatakis et al. 2011; Geva et al. 2012). Thus, in the wake of preservation of behavioral performance, as in our older participants, RIFG activations may be related to overall task performance and/or its nonlinguistic aspects.

As per our second hypothesis, we expected to find higher connectivity of left hemispheric areas, specifically the LINS and LIFG, as a mechanism of preserving phonological function in older adults. We found significant connectivity of LIFG with bilateral frontal and temporal areas in the older cohort and no significant connectivity in the younger cohort. Interestingly, LIFG connected in a more bilateral fashion in the older participants, to both left frontotemporal areas as well as the right posterior regions. Although we were unable to detect significant group differences in connectivity, the finding of significant bilateral connectivity in the older group and lack of significant connectivity in the younger group, lends some support to our hypothesis regarding efficient cognitive processing from LIFG in older adults. Also, although the subtractive analysis between older and younger adults did not show a significant difference at the FWE corrected threshold, connectivity was higher in the older group compared with the younger group at an uncorrected threshold of *P* < 0.001. However, we were unable to demonstrate correlation of these interactions with cognitive performance in older or younger adults, which may partly be an issue of statistical power. The error rates were very low in both groups creating a ceiling effect, which limits our ability to detect a meaningful correlation with this behavioral measure directly. Continuing to revisit our second hypothesis relating to connectivity of left hemispheric regions, we were able to demonstrate that the LINS connected bilaterally in the older adults and showed no significant connectivity in younger adults, and interestingly did so to bilateral inferior frontal regions.

Using a parametric RT model applied to the PPI analysis, we showed that functional connectivity between the LINS and Left frontal regions correlates negatively with RT and thus related to better behavioral performance. It is also noteworthy that connectivity between LINS and LIFG correlated with lower error rates further, supporting the idea of preserved left frontotemporal networks being involved in phonological processing. However, the latter finding in relation to error rates needs to be interpreted cautiously given the low margin for statistical significance and the number of variables used in our correlation analysis. Given that behavioral performance of both groups was similar (no significant difference in RTs or error rates between the two groups, as shown above) and left insular connectivity was higher in older adults, it would be reasonable to, at least, speculate the left hemispheric regions connect more efficiently to preserve function. This idea is further reinforced by the negative relationship between DTI and functional connectivity in keeping with the “less wiring, more firing” hypothesis (Daselaar et al. 2015).

Higher left frontotemporal connectivity from LIFG and LINS for the words > baseline contrast in older adults suggests that interactions of these left hemispheric regions may support core linguistic components during phonological processing. This is in keeping with left hemisphere predominance of other aspects of phonological processing at the network level (Glasser and Rilling 2008; Price 2010; Xiang et al. 2010).

As per our third hypothesis, we expected to find loss of white matter tracts in the older group. This was indeed the case with our DTI analysis, which revealed lower FA in the older group in bilateral corona radiata and the genu of the corpus callosum pointing to the involvement of interhemispheric tracts. Since our cohort of older adults had equivalent task performance to younger participants, the connectivity changes that we describe with our first two hypotheses above are likely to underlie this functional preservation despite loss of interhemispheric white matter integrity. This is supported by the fact that DTI measures did not relate to error rates or reaction times while inversely relating to connectivity measures from the insula to the LIFG (Fig. 2). Loss of white matter in the corpus callosum has been linked to poorer performance on unimanual and bimanual tasks (Basso et al. 1987; Fling et al. 2011a,b). Thus, at the functional connectivity level, particularly in reference to the LINS, we found evidence to support compensation in the HAROLD model.

Recent work on resting functional connectivity changes with aging has shown that decreased intranetwork connectivity is associated with loss of behavioral performance, whereas decreased internetwork connectivity does not impact on behavior in older adults, lending support to decreased specificity (Wierenga et al. 2008; Stamatakis et al. 2011; Antonenko and Flöel 2014; Geerligs et al. 2014) and segregation of functional networks with age (Oldfield 1971; Chan et al. 2014). Our data support this idea and importantly, demonstrates that despite loss of structural integrity of interhemispheric white matter tracts, functional network changes are able to preserve cognitive function. This is a particularly important finding and is in keeping with emerging literature (Daselaar et al. 2015) looking at the mechanisms of higher functional activation in older adults who show loss of white matter tracts. This relationship merits investigation in other cognitive domains. Regarding functional connectivity per se, in the memory domain, a shift from right prefrontal to bilateral connectivity has been shown to preserve recall function in older adults (Cabeza et al. 1997). Grady et al. (2003) demonstrated a ventral to dorsal shift in hippocampal connectivity to preserve memory encoding in older adults. A differential effect on functional connectivity with aging has also been shown in the motor domain (Rowe et al. 2006). How these various functional connectivity changes map to loss of specific white matter tracts remains to be clarified.

Our study has a few limitations. We did not map individual DTI scans to the functional scans. However, both sets of images were mapped to standard atlas‐based space and were acquired one after the other in the same session. We also acknowledge that the seed regions that we chose are likely to be heteromodal (Friederici 2011), thus subserving a multitude of cognitive functions. However, with PPI analysis the functional network interactions can be reliably explored in the context of the specific psychological process being examined by the task, which would allow us to make the inferences that we present (Friston et al. 1997).

Furthermore, our analysis of the DTI data provides information about loss of white matter at certain loci as we describe, but does not explore specific white matter tracts that are crucial for specific language domains (Catani et al. 2005; Friederici 2009). Our DTI protocol was developed for application in healthy adults as well as patient populations alongside fMRI acquisition. This required optimization of scanning time, alongside achieving a high contrast‐to‐noise ratio. Use of multiple b‐values allows this to happen (Correia et al. 2011) with a limited directional acquisition. While we were able to obtain robust anisotropy measures, we were unable to do a tractography analysis with our data (Correia et al. 2009). Future work with tractography‐based analysis would be able to further elicit detailed anatomical – functional relationships in aging and their correspondence to specific language domains.

Another potential issue relates to the difficulty of the task we employed to assess phonological retrieval. It is possible that a task with higher cognitive load may have uncovered deficits in the older adults, as shown in previous work (Stern et al. 2012). The very low error rates in our task is likely to have created a ceiling effect, thus limiting our ability to detect a direct relationship between behavioral measures and functional connectivity. However, our primary aim was to find physiological substrates of preserved cognition rather than those involved in functional decline.

Our findings in the LIFG for the older group support a shift from ipsilateral to bilateral connectivity with healthy aging. Similarly, the left insula showed greater connectivity to bilateral inferior frontal regions in the older subjects when compared with to the younger group. We also showed that connectivity of left insula to left frontal regions correlated with behavioral performance on application of a RT parametric model to PPI connectivity measures. This is in keeping with the emerging idea that core language‐processing systems are not distinct between similarly performing younger and older adults (Shafto and Tyler 2014).

Another important limitation is the fact that the number of years of education was not matched between the two age groups. However, the younger group had a higher number of total education years, which would potentially bias the results against the hypothesis. Despite this limitation, our older cohort had higher functional connectivity from LINS and had a statistical trend to higher connectivity from the LIFG. It is, thus, conceivable that in an older cohort with a higher number of education years, may have uncovered a stronger relationship in the same direction.

It is interesting to note that preservation of syntactic function, considered to be a strongly left lateralized function in language, is closely related to preserved left‐sided frontotemporal connectivity in left hemisphere‐damaged individuals (Shafto et al. 2007; Tyler et al. 2010b; Papoutsi et al. 2011). Also, left hemisphere virtual lesions trigger adaptive plasticity in the right hemisphere homologs (Hartwigsen et al. 2013). Our findings suggest that left hemispheric connectivity drives preserved language function with age. This raises the possibility that age‐related neuroplasticity may be different from that arising from focal brain lesions and age needs to be considered in exploration of mechanism based restorative therapies. Adaptive neuroplasticity may also depend on the level of baseline cognitive function in older adults (Christensen et al. 1999; Schiavone et al. 2009).

## Conclusion

Overall our findings suggest that while key left hemisphere language areas connect bihemispherically with age, despite loss of structural connectivity between cortical areas. These functional network changes may have a role in preserving function. On other hand, right hemispheric homologs that do activate with aging do not drive preserved cognition. This has implications in understanding of not just age‐related neuroplasticity of language, but also recovery of the brain function from focal lesions such as stroke that have a predilection for age and development of restorative strategies.

## Conflict of Interest

None declared.

## Supporting information


**Table S1.** PPI from RIFG at a cluster threshold, *P* < 0.05 (FWE corrected) and clusters with ≥20 voxels.
**Table S2.** PPI from LIFG at a cluster threshold 0.05 (FWE corrected) and clusters with ≥20 voxels.
**Table S3.** PPI from LINS −30,20,−14 at a cluster threshold 0.05 (FWE corrected) and clusters with ≥20 voxels.

## References

[brb3495-bib-0001] Aboitiz, F. 1992 Brain connections: interhemispheric fiber systems and anatomical brain asymmetries in humans. Biol. Res. 25:51–61. 1365702

[brb3495-bib-0002] Antonenko, D. , and A. Flöel . 2014 Healthy aging by staying selectively connected: a mini‐review. Gerontology 60:3–9. 24080587 10.1159/000354376

[brb3495-bib-0003] Ashburner, J. , and K. J. Friston . 2005 Unified segmentation. NeuroImage 26:839–851. 15955494 10.1016/j.neuroimage.2005.02.018

[brb3495-bib-0004] Basso, A. , E. Capitani , and M. Laiacona . 1987 Raven's coloured progressive matrices: normative values on 305 adult normal controls. Funct. Neurol. 2:189–194. 3666548

[brb3495-bib-0005] Cabeza, R. 2002 Hemispheric asymmetry reduction in older adults: the HAROLD model. Psychol. Aging 17:85–100. 11931290 10.1037//0882-7974.17.1.85

[brb3495-bib-0006] Cabeza, R. , A. R. McIntosh , E. Tulving , L. Nyberg , and C. L. Grady . 1997 Age‐related differences in effective neural connectivity during encoding and recall. NeuroReport 8:3479–3483. 9427311 10.1097/00001756-199711100-00013

[brb3495-bib-0007] Catani, M. , D. K. Jones , and D. H. Ffytche . 2005 Perisylvian language networks of the human brain. Ann. Neurol. 57:8–16. 15597383 10.1002/ana.20319

[brb3495-bib-0008] Cereda, C. , J. Ghika , P. Maeder , and J. Bogousslavsky . 2002 Strokes restricted to the insular cortex. Neurology 59:1950–1955. 12499489 10.1212/01.wnl.0000038905.75660.bd

[brb3495-bib-0009] Chan, M. Y. , D. C. Park , N. K. Savalia , S. E. Petersen , and G. S. Wig . 2014 Decreased segregation of brain systems across the healthy adult lifespan. Proc. Natl Acad. Sci. USA 111:E4997–E5006. 25368199 10.1073/pnas.1415122111 PMC4246293

[brb3495-bib-0010] Christensen, H. , A. J. Mackinnon , A. E. Korten , A. F. Jorm , A. S. Henderson , P. Jacomb , et al. 1999 An analysis of diversity in the cognitive performance of elderly community dwellers: individual differences in change scores as a function of age. Psychol. Aging 14:365–379. 10509693 10.1037//0882-7974.14.3.365

[brb3495-bib-0011] Correia, M. M. , T. A. Carpenter , and G. B. Williams . 2009 Looking for the optimal DTI acquisition scheme given a maximum scan time: are more b‐values a waste of time? Magn. Reson. Imaging 27:163–175. 18687552 10.1016/j.mri.2008.06.011

[brb3495-bib-0012] Correia, M. M. , V. F. J. Newcombe , and G. B. Williams . 2011 Contrast‐to‐noise ratios for indices of anisotropy obtained from diffusion MRI: a study with standard clinical b‐values at 3T. NeuroImage 57:1103–1115. 21392583 10.1016/j.neuroimage.2011.03.004

[brb3495-bib-0013] Daselaar, S. M. , V. Iyengar , S. W. Davis , K. Eklund , S. M. Hayes , and R. E. Cabeza . 2015 Less wiring, more firing: low‐performing older adults compensate for impaired white matter with greater neural activity. Cereb. Cortex 25:983–990. 24152545 10.1093/cercor/bht289 PMC4366614

[brb3495-bib-0014] Davis, S. W. , J. E. Kragel , D. J. Madden , and R. Cabeza . 2011 The architecture of cross‐hemispheric communication in the aging brain: linking behavior to functional and structural connectivity. Cereb. Cortex 22:232–242. 21653286 10.1093/cercor/bhr123 PMC3236798

[brb3495-bib-0015] Fling, B. W. , and R. D. Seidler . 2012 Fundamental differences in callosal structure, neurophysiologic function, and bimanual control in young and older adults. Cereb. Cortex 22:2643–2652. 22166764 10.1093/cercor/bhr349 PMC3464417

[brb3495-bib-0016] Fling, B. W. , S. J. Peltier , J. Bo , R. C. Welsh , and R. D. Seidler . 2011a Age differences in interhemispheric interactions: callosal structure, physiological function, and behavior. Front. Neurosci. 5:38. 21519384 10.3389/fnins.2011.00038 PMC3077973

[brb3495-bib-0017] Fling, B. W. , C. M. Walsh , A. S. Bangert , P. A. Reuter‐Lorenz , R. C. Welsh , and R. D. Seidler . 2011b Differential callosal contributions to bimanual control in young and older adults. J. Cogn. Neurosci. 23:2171–2185. 20954936 10.1162/jocn.2010.21600 PMC3809031

[brb3495-bib-0018] Fox, M. D. , and M. E. Raichle . 2007 Spontaneous fluctuations in brain activity observed with functional magnetic resonance imaging. Nat. Rev. Neurosci. 8:700–711. 17704812 10.1038/nrn2201

[brb3495-bib-0019] Friederici, A. D. 2009 Pathways to language: fiber tracts in the human brain. Trends Cogn. Sci. 13:175–181. 19223226 10.1016/j.tics.2009.01.001

[brb3495-bib-0020] Friederici, A. D. 2011 The brain basis of language processing: from structure to function. Physiol. Rev. 91:1357–1392. 22013214 10.1152/physrev.00006.2011

[brb3495-bib-0021] Friederici, A. D. , and S. M. Gierhan . 2013 The language network. Curr. Opin. Neurobiol. 23:250–254. 23146876 10.1016/j.conb.2012.10.002

[brb3495-bib-0022] Friederici, A. D. , J. Brauer , and G. Lohmann . 2011 Maturation of the language network: from inter‐ to intrahemispheric connectivities. Ed. Antoni Rodriguez‐Fornells. PLoS One 6:e20726. 21695183 10.1371/journal.pone.0020726 PMC3113799

[brb3495-bib-0023] Friston, K. J. , C. Buechel , G. R. Fink , J. Morris , E. Rolls , and R. J. Dolan . 1997 Psychophysiological and modulatory interactions in neuroimaging. NeuroImage 6:218–229. 9344826 10.1006/nimg.1997.0291

[brb3495-bib-0024] Galdo‐Alvarez, S. , M. Lindín , and F. Díaz . 2009 The effect of age on event‐related potentials (ERP) associated with face naming and with the tip‐of‐the‐tongue (TOT) state. Biol. Psychol. 81:14–23. 19428964 10.1016/j.biopsycho.2009.01.002

[brb3495-bib-0025] Geerligs, L. , N. M. Maurits , R. J. Renken , and M. M. Lorist . 2014 Reduced specificity of functional connectivity in the aging brain during task performance. Hum. Brain Mapp. 35:319–330. 22915491 10.1002/hbm.22175 PMC6869200

[brb3495-bib-0026] Geva, S. , P. S. Jones , J. T. Crinion , C. J. Price , J.‐C. Baron , and E. A. Warburton . 2011a The neural correlates of inner speech defined by voxel‐based lesion‐symptom mapping. Brain 134:3071–3082. 21975590 10.1093/brain/awr232 PMC3187541

[brb3495-bib-0027] Geva, S. , S. Bennett , E. A. Warburton , and K. Patterson . 2011b Discrepancy between inner and overt speech: implications for post‐stroke aphasia and normal language processing. Aphasiology 25:323–343.

[brb3495-bib-0028] Geva, S. , P. S. Jones , J. T. Crinion , C. J. Price , J.‐C. Baron , and E. A. Warburton . 2012 The effect of aging on the neural correlates of phonological word retrieval. J. Cogn. Neurosci. 24:2135–2146. 22849403 10.1162/jocn_a_00278 PMC3477855

[brb3495-bib-0029] Glasser, M. F. , and J. K. Rilling . 2008 DTI tractography of the human brain's language pathways. Cereb. Cortex 18:2471–2482. 18281301 10.1093/cercor/bhn011

[brb3495-bib-0030] Goh, J. O. S. 2011 Functional dedifferentiation and altered connectivity in older adults: neural accounts of cognitive aging. Aging Dis. 2:30–48. 21461180 PMC3066008

[brb3495-bib-0031] Grady, C. L. 2008 Cognitive neuroscience of aging. Ann. N. Y. Acad. Sci. 1124:127–144. 18400928 10.1196/annals.1440.009

[brb3495-bib-0032] Grady, C. L. , A. R. McIntosh , and F. I. M. Craik . 2003 Age‐related differences in the functional connectivity of the hippocampus during memory encoding. Hippocampus 13:572–586. 12921348 10.1002/hipo.10114

[brb3495-bib-0033] Hagoort, P. 2013 MUC (memory, unification, control) and beyond. Front. Psychol. 4:416. 23874313 10.3389/fpsyg.2013.00416 PMC3709422

[brb3495-bib-0034] Hartwigsen, G. , D. Saur , C. J. Price , S. Ulmer , A. Baumgaertner , and H. R. Siebner . 2013 Perturbation of the left inferior frontal gyrus triggers adaptive plasticity in the right homologous area during speech production. Proc. Natl Acad. Sci. USA 110:16402–16407. 24062469 10.1073/pnas.1310190110 PMC3799383

[brb3495-bib-0035] Horwitz, B. 2003 The elusive concept of brain connectivity. NeuroImage 19:466–470. 12814595 10.1016/s1053-8119(03)00112-5

[brb3495-bib-0036] Kasahara, M. , D. K. Menon , C. H. Salmond , J. G. Outtrim , J. V. Taylor Tavares , T. A. Carpenter , et al. 2010 Altered functional connectivity in the motor network after traumatic brain injury. Neurology 75:168–176. 20625170 10.1212/WNL.0b013e3181e7ca58 PMC2905928

[brb3495-bib-0037] Kasahara, M. , D. K. Menon , C. H. Salmond , J. G. Outtrim , J. V. T. Tavares , T. A. Carpenter , et al. 2011 Traumatic brain injury alters the functional brain network mediating working memory. Brain Inj. 25:1170–1187. 21932994 10.3109/02699052.2011.608210

[brb3495-bib-0038] Kerchner, G. A. , C. A. Racine , S. Hale , R. Wilheim , V. Laluz , B. L. Miller , et al. 2012 Cognitive processing speed in older adults: relationship with white matter integrity. Ed. Emmanuel Andreas Stamatakis. PLoS One 7:e50425. 23185621 10.1371/journal.pone.0050425 PMC3503892

[brb3495-bib-0039] Lebel, C. , M. Gee , R. Camicioli , M. Wieler , W. Martin , and C. Beaulieu . 2012 Diffusion tensor imaging of white matter tract evolution over the lifespan. NeuroImage 60:340–352. 22178809 10.1016/j.neuroimage.2011.11.094

[brb3495-bib-0040] Meinzer, M. , T. Flaisch , L. Wilser , C. Eulitz , B. Rockstroh , T. Conway , et al. 2009 Neural signatures of semantic and phonemic fluency in young and old adults. J. Cogn. Neurosci. 21:2007–2018. 19296728 10.1162/jocn.2009.21219 PMC2730979

[brb3495-bib-0041] Nusbaum, A. O. , C. Y. Tang , M. S. Buchsbaum , T. C. Wei , and S. W. Atlas . 2001 Regional and global changes in cerebral diffusion with normal aging. AJNR Am. J. Neuroradiol. 22:136–142. 11158899 PMC7975529

[brb3495-bib-0042] Oldfield, R. C. 1971 The assessment and analysis of handedness: the Edinburgh inventory. Neuropsychologia 9:97–113. 5146491 10.1016/0028-3932(71)90067-4

[brb3495-bib-0043] Papoutsi, M. , E. A. Stamatakis , J. Griffiths , W. D. Marslen‐Wilson , and L. K. Tyler . 2011 Is left fronto‐temporal connectivity essential for syntax? Effective connectivity, tractography and performance in left‐hemisphere damaged patients. NeuroImage 58:656–664. 21722742 10.1016/j.neuroimage.2011.06.036

[brb3495-bib-0044] Passamonti, L. , J. B. Rowe , C. Schwarzbauer , M. P. Ewbank , E. von dem Hagen , and A. J. Calder . 2009 Personality predicts the brain's response to viewing appetizing foods: the neural basis of a risk factor for overeating. J. Neurosci. 29:43–51. 19129383 10.1523/JNEUROSCI.4966-08.2009 PMC6664921

[brb3495-bib-0045] Poeppel, D. , and G. Hickok . 2004 Towards a new functional anatomy of language. Cognition 92:1–12. 15037124 10.1016/j.cognition.2003.11.001

[brb3495-bib-0046] Price, C. J. 2010 The anatomy of language: a review of 100 fMRI studies published in 2009. Ann. N. Y. Acad. Sci. 1191:62–88. 20392276 10.1111/j.1749-6632.2010.05444.x

[brb3495-bib-0047] Rijntjes, M. , C. Weiller , T. Bormann , and M. Musso . 2012 The dual loop model: its relation to language and other modalities. Front. Evol. Neurosci. 4:9. 22783188 10.3389/fnevo.2012.00009 PMC3388276

[brb3495-bib-0048] Rowe, J. B. , H. Siebner , S. R. Filipovic , C. Cordivari , W. Gerschlager , J. Rothwell , et al. 2006 Aging is associated with contrasting changes in local and distant cortical connectivity in the human motor system. NeuroImage 32:747–760. 16797190 10.1016/j.neuroimage.2006.03.061

[brb3495-bib-0049] Salthouse, T. A. 2010 Selective review of cognitive aging. J. Int. Neuropsychol. Soc. 16:754–760. 20673381 10.1017/S1355617710000706 PMC3637655

[brb3495-bib-0050] Schiavone, F. , R. A. Charlton , T. R. Barrick , R. G. Morris , and H. S. Markus . 2009 Imaging age‐related cognitive decline: a comparison of diffusion tensor and magnetization transfer MRI. J. Magn. Reson. Imaging 29:23–30. 19097099 10.1002/jmri.21572

[brb3495-bib-0051] Shafto, M. A. , and L. K. Tyler . 2014 Language in the aging brain: the network dynamics of cognitive decline and preservation. Science 346:583–587. 25359966 10.1126/science.1254404

[brb3495-bib-0052] Shafto, M. A. , D. M. Burke , E. A. Stamatakis , P. P. Tam , and L. K. Tyler . 2007 On the tip‐of‐the‐tongue: neural correlates of increased word‐finding failures in normal aging. J. Cogn. Neurosci. 19:2060–2070. 17892392 10.1162/jocn.2007.19.12.2060 PMC2373253

[brb3495-bib-0053] Shafto, M. A. , E. A. Stamatakis , P. P. Tam , and L. K. Tyler . 2010 Word retrieval failures in old age: the relationship between structure and function. J. Cogn. Neurosci. 22:1530–1540. 19642890 10.1162/jocn.2009.21321

[brb3495-bib-0054] Stamatakis, E. A. , M. A. Shafto , G. Williams , P. Tam , and L. K. Tyler . 2011 White matter changes and word finding failures with increasing age. PLoS One 6:e14496. 21249127 10.1371/journal.pone.0014496 PMC3017545

[brb3495-bib-0055] Stern, Y. , B. C. Rakitin , C. Habeck , Y. Gazes , J. Steffener , A. Kumar , et al. 2012 Task difficulty modulates young–old differences in network expression. Brain Res. 1435:130–145. 22197699 10.1016/j.brainres.2011.11.061 PMC3406734

[brb3495-bib-0056] Tyler, L. K. , M. A. Shafto , B. Randall , P. Wright , W. D. Marslen‐Wilson , and E. A. Stamatakis . 2010a Preserving syntactic processing across the adult life span: the modulation of the frontotemporal language system in the context of age‐related atrophy. Cereb. Cortex 20:352–364. 19505991 10.1093/cercor/bhp105 PMC2803734

[brb3495-bib-0057] Tyler, L. K. , P. Wright , B. Randall , W. D. Marslen‐Wilson , and E. A. Stamatakis . 2010b Reorganization of syntactic processing following left‐hemisphere brain damage: does right‐hemisphere activity preserve function? Brain 133:3396–3408. 20870779 10.1093/brain/awq262 PMC2965424

[brb3495-bib-0058] Upton, C. , and E. Upton . 2004 Oxford rhyming dictionary. Oxford University Press, Oxford, UK.

[brb3495-bib-0059] Vigneau, M. , V. Beaucousin , P.‐Y. Hervé , G. Jobard , L. Petit , F. Crivello , et al. 2011 What is right‐hemisphere contribution to phonological, lexico‐semantic, and sentence processing? NeuroImage 54:577–593. 20656040 10.1016/j.neuroimage.2010.07.036

[brb3495-bib-0060] Wierenga, C. E. , M. Benjamin , K. Gopinath , W. M. Perlstein , C. M. Leonard , L. J. G. Rothi , et al. 2008 Age‐related changes in word retrieval: role of bilateral frontal and subcortical networks. Neurobiol. Aging 29:436–451. 17147975 10.1016/j.neurobiolaging.2006.10.024

[brb3495-bib-0061] Wise, R. J. , J. Greene , C. Büchel , and S. K. Scott . 1999 Brain regions involved in articulation. Lancet 353:1057–1061. 10199354 10.1016/s0140-6736(98)07491-1

[brb3495-bib-0062] Xiang, H. D. , H. M. Fonteijn , D. G. Norris , and P. Hagoort . 2010 Topographical functional connectivity pattern in the perisylvian language networks. Cereb. Cortex 20:549–560. 19546155 10.1093/cercor/bhp119

