# Supplementary material for: Dominant hemisphere functional networks compensate for structural connectivity loss to preserve phonological retrieval with aging
Source: Brain Behav. 2016 Jul 27;6(9):e00495. doi: 10.1002/brb3.495 (PMC5036427; doi:10.1002/brb3.495)
Supplement: Supplementary file 1 — Table S1. PPI from RIFG at a cluster threshold, P < 0.05 (FWE corrected) and clusters with ≥20 voxels. Table S2. PPI from LIFG at a cluster threshold 0.05 (FWE corrected) and clusters with ≥20 voxels. Table S3. PPI from LINS −30,20,−14 at a cluster threshold 0.05 (FWE corrected) and clusters with ≥20 voxels. [file BRB3-6-e00495-s001.docx]

**Supplementary Table 1. PPI from RIFG at a cluster threshold, p< 0.05 (FWE corrected) and clusters with >=20 voxels**

|  | **Seed region** | **Brain region** | **x** | **y** | **z** | **Peak t value** | **Peak z score** | **Error rate vs PPI r (p)** | **Cluster size** |
| --- | --- | --- | --- | --- | --- | --- | --- | --- | --- |
| All | 40, 36, -2 | RIFG POrb | 40 | 34 | -2 | 12.67 | 7.25 |  | 162304(contiguous) |
|  |  | Left middle frontal | -40 | 42 | 20 | 12.20 | 7.19 |  | 162304(contiguous) |
|  |  | LIFG P Tri | -42 | 34 | 16 | 12.20 | 7.19 |  | 162304(contiguous) |
|  |  |  |  |  |  |  |  |  |  |
| Old |  | Left middle frontal | -40 | 42 | 20 | 9.57 | 6.33 | -0.20(0.406) | 129286(contiguous) |
|  |  | Right post central | 20 | -38 | 52 | 9.17 | 6.18 | -0.20(0.424) | 188(contiguous) |
|  |  | RIFG POrb | 40 | 36 | -2 | 9.00 | 6.12 | 0.03(0.917) | 188(contiguous) |
|  |  |  |  |  |  |  |  |  |  |
| Y |  | RIFG PTri | 40 | 34 | 0 | 10.16 | 6.53 | 0.21(0.544) | 64 |
|  |  | LIFG PTri | -42 | 34 | 16 | 9.99 | 6.24 | 0.16(0.631) | 85992(contiguous) |
|  |  | Left middle frontal | -34 | 36 | 18 | 9.27 | 6.22 | -0.33(0.315) | 85992(contiguous) |
|  |  |  |  |  |  |  |  |  |  |
| Y>O |  | RIFG PTri | 40 | 32 | 0 | 4.83 | 4.09 | 0.32(0.087) | 788(contiguous) |
|  |  | RIFG PTri | 52 | 40 | 6 | 3.89 | 3.45 |  | 788(contiguous) |
|  |  | RIFG POp | 60 | 22 | 20 | 3.89 | 3.45 | 0.01(0.981) | 788(contiguous) |
|  |  | LIFG PTri | -36 | 34 | 18 | 4.74 | 4.03 | 0.14(0.475) | 936(contiguous) |
|  |  | LIFG POp | -56 | 10 | 26 | 3.87 | 3.44 | -0.01(0.977) | 936 (contiguous) |
|  |  | Left rolandic operculum | -58 | 6 | 8 | 3.73 | 3.33 | 0.25(0.184) | 936(contiguous) |

**Supplementary Table 2. PPI from LIFG at a cluster threshold 0.05 (FWE corrected) and clusters with >=20 voxels**

|  | **Seed Region** | **Brain region** | **x** | **y** | **z** | **Peak t value** | **Peak z score** | **Error rate vs PPI r (p)** | **Cluster size** |
| --- | --- | --- | --- | --- | --- | --- | --- | --- | --- |
| All | -46,26,8 | RIFG POrb | 46 | 48 | -4 | 4.77 | 4.07 |  | 451 |
|  |  | Cluster to RIFG (P opercularis) | 24 | -10 | 34 | 4.15 | 3.65 |  | 5510 |
|  |  | Cluster to LIFG (P opercularis) | -28 | 6 | 28 | 4.08 | 3.60 |  | 595 |
|  |  | Left middle frontal | -24 | 40 | -16 | 4.35 | 3.79 |  | 671(contiguous) |
|  |  | Cluster to L middle frontal | -26 | 42 | 0 | 3.81 | 3.40 |  |  |
|  |  | LIFG PTri | -36 | 40 | 4 | 3.78 | 3.98 |  | 671(contiguous) |
|  |  |  |  |  |  |  |  |  |  |
| Old |  | RIFG POrb | 46 | 44 | -4 | 5.47 | 4.14 | 0.22(0.374) | 1980(contiguous) |
|  |  | RIFG PTri | 44 | 36 | 28 | 5.18 | 4.00 | 0.11(0.642) | 1980(contiguous) |
|  |  | RIFG PTri | 42 | 28 | 24 | 4.99 | 3.91 |  |  |
|  |  | Lt middle frontal (orb) | -40 | 52 | -6 | 5.23 | 4.03 | 0.04(0.872) | 691(contiguous) |
|  |  | LIFG POrb | -48 | 44 | -2 | 4.46 | 3.61 | 0.11(0.648) | 691(contiguous) |
|  |  | Rt cerebellum | 16 | -86 | -28 | 4.65 | 3.72 | 0.05(0.842) | 318(contiguous) |
|  |  | Rt cerebellum | 16 | -70 | -26 | 3.89 | 3.27 |  |  |
|  |  | Rt inferior temporal | 46 | -54 | -22 | 4.99 | 3.57 | 0.05(0.851) | 434 |
|  |  | Lt thalamus | -2 | -12 | 6 | 4.59 | 3.69 | 0.32(0.177) | 1062(contiguous) |
|  |  | Rt precentral | 50 | 4 | 48 | 3.92 | 3.29 | -0.03(0.916) | 1062(contiguous) |
|  |  | Rt Caudate | 18 | -8 | 22 | 3.81 | 3.22 | -0.06(0.824) | 1062(contiguous) |
|  |  |  |  |  |  |  |  |  |  |
| Y |  | None at above threshold |  |  |  |  |  |  |  |
|  | At p<0.001 | Left insula | -32 | 10 | 16 | 4.43 | 3.22 | -0.13(0.715) | 38 |
|  | At p<0.001 | Cluster to LIFG P opercularis | -24 | 12 | 34 | 4.29 | 3.13 | -0.16(0.643) | 66 |
|  | At p<0.001 | Left middle frontal orbitalis | -28 | 42 | -20 | 4.01 | 3.02 | -0.17(0.626) | 48 |
|  | At p<0.001 | Left sup medial frontal | -10 | 58 | 30 | 4.00 | 3.02 | -0.18(0.606) |  |
|  |  |  |  |  |  |  |  |  |  |
| Y>O |  | None at above or p<0.001 |  |  |  |  |  |  |  |
|  |  |  |  |  |  |  |  |  |  |
| O>Y |  | None at above or p<0.001 |  |  |  |  |  |  |  |
|  |  |  |  |  |  |  |  |  |  |

**Supplementary Table 3. PPI from LINS -30,20,-14 at a cluster threshold 0.05 (FWE corrected) and clusters with >=20 voxels**

|  | **Seed Region** | **Brain region** | **x** | **y** | **z** | **Peak t value** | **Peak z score** | **Error rate vs PPI r (p)** | **Cluster size** |
| --- | --- | --- | --- | --- | --- | --- | --- | --- | --- |
| All | -30,20,-14 | None at above threshold |  |  |  |  |  |  |  |
|  |  |  |  |  |  |  |  |  |  |
| Old |  | LIFG PTri | -42 | 38 | 2 | 5.78 | 4.29 | -0.11(0.644) | 712(contiguous) |
|  |  | **Left middle frontal** | -38 | 58 | 6 | 5.76 | 4.28 | **-0.42(0.07)** | 712(contiguous) |
|  |  | LIFG POrb | -40 | 38 | -10 | 4.79 | 3.80 | -0.37(0.100) | 712(contiguous) |
|  |  | Rt middle frontal orb | 38 | 58 | -2 | 5.30 | 4.06 | -0.01(0.983) | 452 |
|  |  | Rt sup frontal orb | 20 | 20 | -12 | 4.49 | 3.69 | 0.19(0.437) | 222(contiguous) |
|  |  | Rt caudate | 12 | 26 | -6 | 3.85 | 3.25 | 0.27(0.275) | 222(contiguous) |
|  |  |  |  |  |  |  |  |  |  |
| Y |  | None at above or p<0.001 |  |  |  |  |  |  |  |
|  |  |  |  |  |  |  |  |  |  |
| O>Y |  | None at above threshold |  |  |  |  |  |  |  |
|  | **At p<0.001** | **LIFG POrb** | -44 | 36 | -10 | 5.02 | 4.20 | **-0.40(0.030)** | 517(contiguous) |
|  | **At p<0.001** | Left insula | -34 | 22 | -8 | 3.56 | 3.21 | -0.19(0.320) | 517(contiguous) |
|  | **At p<0.001** | Right middle frontal orbitalis | 48 | 48 | -6 | 4.89 | 4.12 | -0.26(0.167) | 83 |
|  | **At p<0.001** | Left fusiform | -36 | -60 | -16 | 3.33 | 3.03 | -0.03(0.861) | 50(contiguous) |
|  | **At p<0.001** | Left middle frontal | -48 | 42 | 20 | 3.26 | 2.97 | -0.06(0.756) | 50(contiguous) |
|  |  |  |  |  |  |  |  |  |  |
|  |  |  |  |  |  |  |  |  |  |
